# Sensitivity of novel silicate and borate-based glass structures on *in vitro* bioactivity and degradation behaviour

**DOI:** 10.1016/j.ceramint.2017.06.146

**Published:** 2017-10-15

**Authors:** Elena Mancuso, Oana Bretcanu, Martyn Marshall, Kenneth W. Dalgarno

**Affiliations:** a School of Mechanical and Systems Engineering, Newcastle University, Claremont Road, Newcastle upon Tyne, UK; b School of Mechanical Engineering, University of Leeds, Woodhouse Lane, Leeds, UK; c Glass Technology Services Ltd., Sheffield, UK

**Keywords:** Bioceramics, Bioactivity, Degradation, Apatite formation

## Abstract

Three novel glass compositions, identified as NCL2 (SiO_2_-based), NCL4 (B_2_O_3_-based) and NCL7 (SiO_2_-based), along with apatite-wollastonite (AW) were processed to form sintered dense pellets, and subsequently evaluated for their *in vitro* bioactive potential, resulting physico-chemical properties and degradation rate. Microstructural analysis showed the carbonated hydroxyapatite (HCA) precipitate morphology following SBF testing to be composition-dependent. AW and the NCL7 formulation exhibited greater HCA precursor formation than the NCL2 and NCL4-derived pellets. Moreover, the NCL4 borate-based samples showed the highest biodegradation rate; with silicate-derived structures displaying the lowest weight loss after SBF immersion. The results of this study suggested that glass composition has significant influence on apatite-forming ability and also degradation rate, indicating the possibility to customise the properties of this class of materials towards the bone repair and regeneration process.

## 1 Introduction

Since the first proposed glass (currently known as Bioglass®) developed by Hench in 1969, and intended for bone tissue applications [1], [2], bioactive glasses become a class of biomaterials which are still widely investigated [3].

Among inorganic biomaterials, Bioglass^®^ has received great attention for its ability to form a strong bond with soft as well as hard host tissue, resulting in what has been recognised as bioactive behaviour [4]. The concept of bioactivity was introduced by Hench at the beginning of 70's, when he described the bonding of 45S5 bioglass to bone as a process based on the formation of a carbonated hydroxyapatite (HCA) layer on the surface of the material in contact with the host tissue [2]. The development of this glass revolutionised the definition of biomaterial, moving the perspective from inert to a material that, interacting with the human body, is capable to elicit a specific biological response [5].

Around a decade later in Japan Kokubo et al. were the first to synthetize a new glass-ceramic material currently known as apatite-wollastonite [6]. This bioceramic demonstrated excellent mechanical properties and an exceptional ability to form a strong chemical bond with bone tissue [7], [8]. In 1990, Kokubo described the capacity of a material to develop an HA-like layer on its surface, when immersed in a simulated body fluid (SBF) solution, as indicative of its bioactivity [9]. The SBF proposed by Kokubo mimics human blood plasma in terms of pH and ionic concentration and it is the most applied preliminary *in vitro* test to assess the bioactive potential of biomedical materials [10].

Many new bioactive formulations in the silicate, phosphate and borate-based system and with variable types of modifiers have been designed [11], [12], [13]. However, it has been found that small variations in the glass main formulation greatly affect material properties such as degradation rate and bioactive potential [14], [15], [16], [17].

**Fig. 1:**
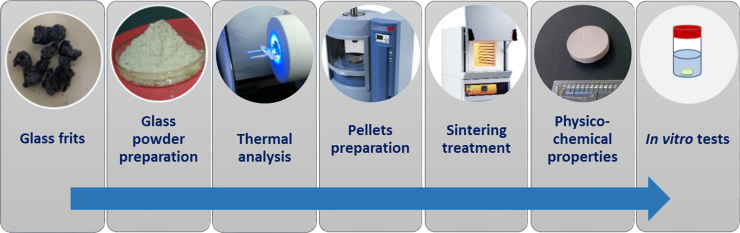
Preparation and characterisation of novel sintered bioceramic pellets.

For the first time, highly complex bioceramic formulations (belonging to the silicate and borate-based glass family), containing different doping agents (*i.e.* MgO, MnO_2_, Al_2_O_3_, CaF_2_, Fe_2_O_3_, ZnO, CuO, Cr_2_O_3_) and in diverse weight percentages, were produced *via* a melting-quenching method [19] and demonstrated good potential as biomaterials for bone tissue applications. Following the desirable requirement to develop materials with sufficient bioactivity and controllable degradation behaviour, the motivation of the present work was to evaluate the effect of these novel bioceramic compositions on apatite-forming ability and degradation rate upon immersion in simulated body fluid (SBF) solution. The novel materials, processed in form of dense sintered pellets (see Fig. 1), were investigated in terms of crystallinity by X-ray diffraction (XRD) and morphological structure through scanning electron microscopy (SEM) before and after 28 days immersion in SBF. Furthermore, the chemical composition of the precipitates was assessed by using x-ray photoelectron spectroscopy (XPS) and energy dispersive spectroscopy (EDX). In addition, measurements of the pH variations, degradation behaviour and ion leaching potential were conducted.

## 2 Materials and methods

### 2.1 Preparation of bioceramic samples and characterisation

Three novel bioceramic formulations were investigated in this study, together with AW, selected as a well characterised comparator material (see Table 1). All the glasses were produced and supplied by Glass Technology Service (GTS) Ltd (Sheffield, UK). The glass frits as received were processed to compact pellets, following the same procedure described in [18].

**Table 1:** Composition of the novel glass formulations.

**CODE**	**GLASS COMPOSITION (wt%)**
NCL2	36.90SiO_2_ – 9.70P_2_O_5_ – 1.90B_2_O_3_ – 3.39Na_2_O – 11.48CaO – 3.85K_2_O – 4.41MgO – 2.38MnO_2_ – 6.97Al_2_O_3_ – 2.13CaF_2_ – 10.92Fe_2_O_3_ – 0.41Li_2_O – 1.97MoO_3_ – 1.52SeO_2_ – 2.07Cr_2_O_3_
NCL4	16.28SiO_2_ – 9.63P_2_O_5_ – 37.77B_2_O_3_ – 4.21Na_2_O – 3.80CaO – 6.38K_2_O – 2.73MgO – 5.52ZnO – 7.03SrO – 2.12CaF_2_ – 1.08CuO – 1.95MoO_3_ – 1.51SeO_2_
NCL7	39.96SiO_2_ – 9.46P_2_O_5_ – 12.39Na_2_O – 11.19CaO – 2.50K_2_O – 1.61MgO – 15.44AgO – 2.13TiO_2_ – 4.26Fe_2_O_3_ – 1.06CuO
AW	4.6 MgO – 44.7 – CaO – 34 SiO_2_ – 16.2 P_2_O_5_ – 0.5 CaF_2_

Briefly, bioceramic pellets were prepared by cold pressing raw glass powders in a cylindrical stainless steel mould (diameter 10 mm) using an automatic hydraulic press (Specac-Atlas™ 8T, Specac Ltd., UK). Subsequently, the pressed pellets, also called green bodies, were treated through a sintering process to consolidate their structure. Hence, they were placed in a furnace (Carbolite 1200 CWF, Carbolite GmbH, Germany) and sintered in accordance to data derived from hot stage microscopy analysis [18], as shown in Table 2. Subsequently, XRD analysis was performed on both sintered and un-sintered samples by using a PANalytical X′Pert Pro MPD, powered by a Philips PW3040/60 X-ray generator fitted with an X′Celerator detector. Diffraction data was acquired by exposing powder samples to Cu-K_α_ X-ray radiation, which was supplied with 40 kV and a current of 40 mA. The data were collected over a 2θ range between 5° and 80° (2θ), with a step size equal to 0.0334°, a counting time per step of 200 s using the scanning X′Celerator detector. Phase identification was carried out by means of the PANalytical X′Pert HighScore Plus© software, in conjunction with the ICDD Powder Diffraction File 2 Database (2004), ICDD Powder Diffraction File 4 - Minerals (2014) and the Crystallography Open Database (February 2013; www.crystallography.net).

**Table 2:** Heat treatments for dense pellets.

**CODE**	**SINTERING TREATMENT**
NCL2	10°/min up to 700 °C, hold for 1 h
NCL4	10°/min up to 625 °C, hold for 1 h
NCL7	10°/min up to 625 °C, hold for 1 h
AW	10°/min up to 850 °C, hold for 1 h

### 2.2 *In vitro* bioactivity test in simulated body fluid

In order to assess the bioactive potential of the novel materials, sintered pellets were soaked for 1, 3, 7, 14 and 28 days in SBF, which was prepared following Kokubo's protocol [10].

Each sample was immersed in 10 ml of acellular SBF then incubated at 37 °C. During the incubation time, the SBF solution was replaced every two days to avoid ionic depletion in the SBF due to the precipitation of inorganic salts on the samples’ surface.

At the end of each time interval, the samples were removed from SBF, then gently rinsed with deionised water (Veolia Water Technologies, UK) and dried at room temperature before starting further characterisation.

### 2.3 Sample characterisation

#### 2.3.1 Morphological and compositional analysis

The structural characteristics and chemical composition of the upper surface of the samples were investigated by SEM/EDS (Philips XL30 ESEM FEG, which is fitted with a Rontec Quantax system for the EDS analysis). Before the imaging acquisition, the specimens were sputtered with a thin layer of gold (approximately 10 nm, sputter time 40 s at 40 mA), and afterward analysed. All the images were taken at an operation voltage of 20 kV, and working distance between 5 and 10 mm.

Additionally, to quantitatively evaluate the composition of the precipitates, XPS analysis was performed using Theta Probe (Thermo Scientific, East Grinstead, UK), with a micro-focused AlKa X-ray source (1486.6 eV), operated with a 400 µm spot size (100 W power). Survey spectra were collected at a pass energy of 200 eV, with the spectrometer operated in standard (not angle-resolved) lens mode. The results were expressed as the average of three points of each sample surface.

#### 2.3.2 Weight loss, pH variation, and ionic leaching potential

The pH of the solutions was measured after each time point using a pH meter (Mettler Toledo Ltd., UK), which was calibrated with standard solutions (at pH 4 and 7) every time before use. Moreover, the sample solubility was quantitatively assessed by measuring the weight loss of the immersed pellets after 1, 3, 7, 14 and 28 days of soaking, using an analytical balance (Kern ABT220-5DM), according to the following formula:

$$\mathrm{Weight}\;\mathrm{loss}\left(\%\right)=\frac{M_{\mathrm{bi}}-M_{\mathrm{af}}}{M_{\mathrm{bi}}}\times 100$$

where $M_{\mathrm{bi}}$ is the mass of the sample before the immersion and $M_{\mathrm{af}}$ is the mass of the sample after the immersion. All the results were expressed as average ± standard deviation (SD). Furthermore, in order to evaluate the ionic release potential of each composition, the ion concentration was measured using an inductively coupled plasma optical emission spectrometer Specto-Ciros-Vision (Sheffield University, UK), which allows simultaneous multi-element analysis following the calibration of the instrument by introduction of standards of known concentrations of the elements of interest.

## 3 Results and discussion

### 3.1 Sintering and crystal structure evolution

**Fig. 2:**
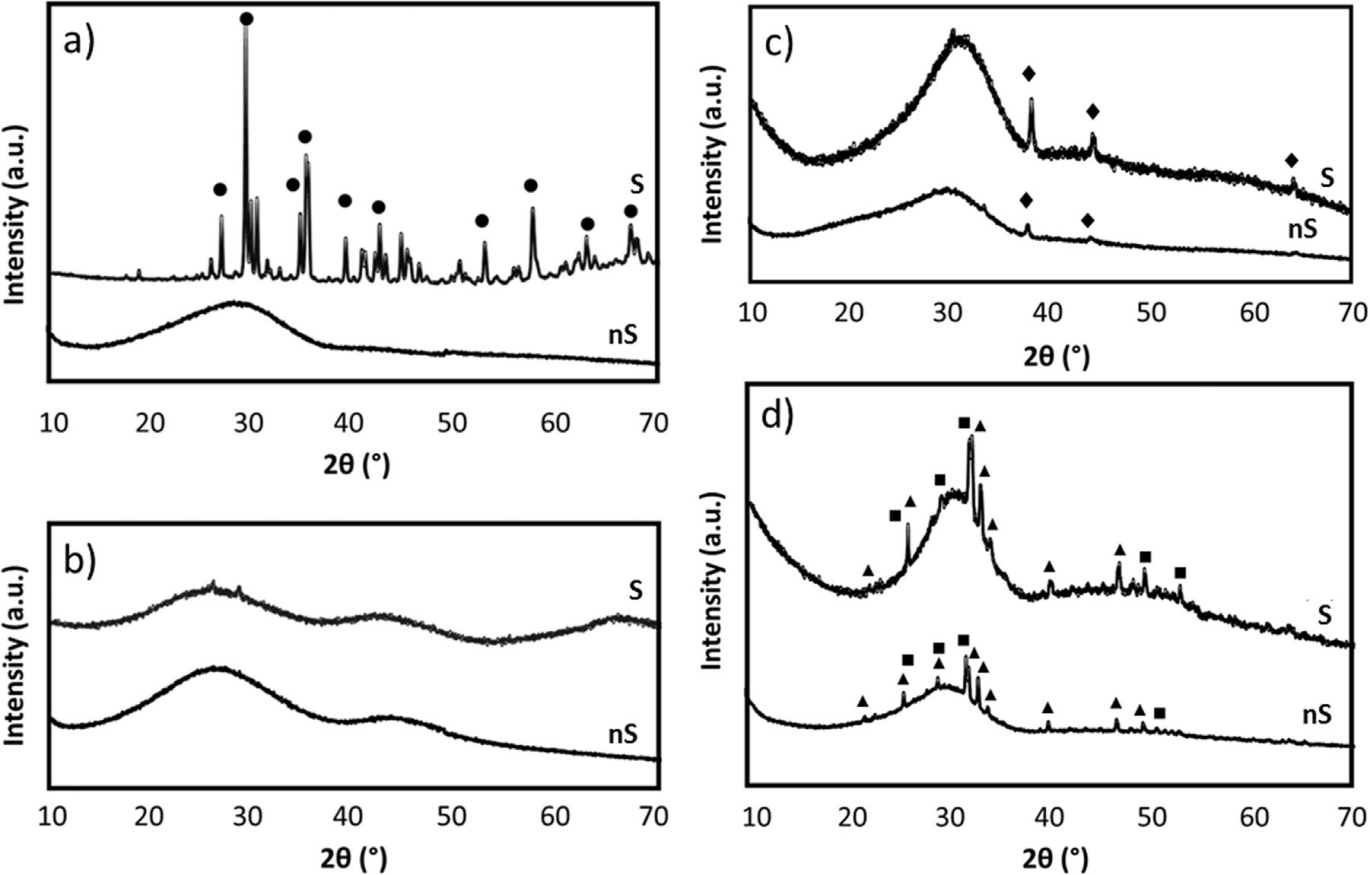
XRD patterns of: a) NCL2, b) NCL4, c) NCL7 and d) AW before sintering (nS) and after sintering (S). • diopside, ♦ silver, ▲ hydroxylapatite, and ■ β-wollastonite.

Sintered bioceramic pellets were successfully fabricated following the heating treatment reported in Table 2, and based on the results previously derived from HSM analysis [19]. Sintering temperatures, which usually range from 30% to 90% of the melting temperature [20], have been found to greatly depend on the material compositions [19] and HSM revealed a powerful technique to identify the optimal sintering intervals of the novel bioceramics. By comparing the XRD patterns of the glass powders with those of the sintered bioceramic pellets (see Fig. 2) we can observe that:

• The post sintering XRD pattern for NCL2 silicate-based glass revealed the presence of a crystalline phase identified as diopside (ICDD ref. code 01-073-6374). Diopside is a Mg-containing compound, which has already been investigated as a biomaterial for bone repair in powder and dense bulk ceramic forms [21]. Furthermore, diopside-derived scaffolds were found to possess good and stable mechanical properties upon immersion in physiological solution due to their low degradation rate [22].
• The XRD analysis confirmed the amorphous nature of NCL4 formulation even after sintering.
• The thermal process did not affect the crystallinity of NCL7 glass-ceramic, which still showed a crystalline phase corresponding to pure silver (ICDD ref. code 04-003-1425).
• The XRD patterns of AW samples revealed the same crystalline phases (hydroxylapatite (ICDD ref. code 01-073-1731) complemented with β-wollastonite (ICDD ref. code 01-071-0880)) before and after the sintering process, confirming the glass-ceramic nature of this formulation [23].

### 3.2 Morphological analysis

**Fig. 3:**
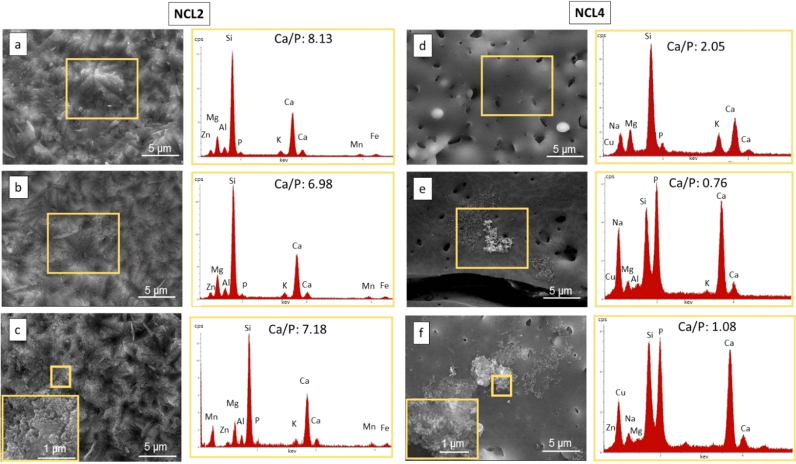
On the left, morphological (5Kx mag) and compositional analysis (at%) of NCL2 bioceramic pellet: a) before immersion in SBF, b) after 7 days and c) after 28 days of immersion in SBF, with the inset showing the precipitate morphology (10Kx). On the right, morphological (5Kx mag) and compositional analysis (at%) of NCL7 bioceramic pellet: d) before immersion in SBF, e) after 7 days and f) after 28 days of immersion in SBF, with the inset showing the precipitate morphology (10Kx).

**Fig. 4:**
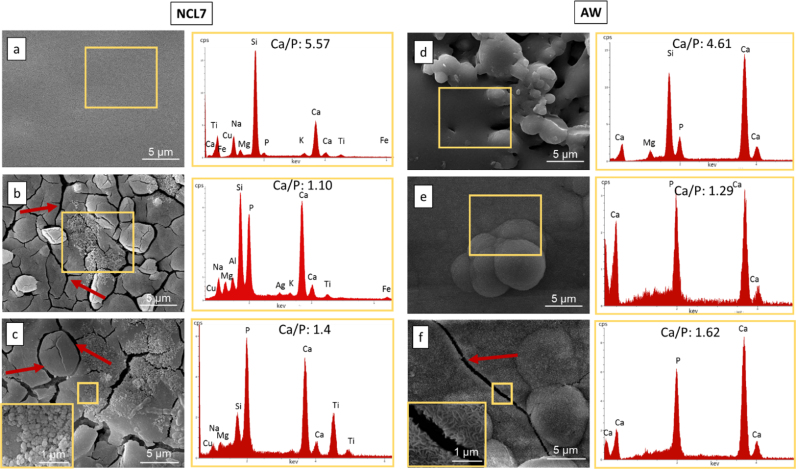
On the left, morphological (5Kx mag) and compositional analysis (at%) of NCL7 bioceramic pellet: a) before immersion in SBF, b) after 7 days and c) after 28 days of immersion in SBF, with the inset showing the precipitate morphology (10Kx). On the right, morphological (5Kx mag) and compositional analysis (at%) of AW bioceramic pellet: d) before immersion in SBF, e) after 7 days and f) after 28 days of immersion in SBF, with the inset showing the precipitate morphology (10Kx). The red arrows indicate the micro-cracks formation on the pellet surface.

The bioactivity of the sintered pellets before and after 7 and 28 days in immersion in SBF solution was firstly evaluated through SEM and EDX analysis, whose outcomes are reported in Fig. 3, Fig. 4.

Of particular interest is the difference in behaviour of the two silicate based formulations. No apatite nucleation was detected for NCL2-based pellets (see Fig. 3(a)–(c)), which after 28 days in immersion developed a homogeneous rough layer onto the sample surface (Fig. 3(c)). Globular shaped agglomerates developed on NCL7 specimens (Fig. 4(a)–(c)), with a consequent increase in the Ca/P ratio from 1.1 at day 7 to 1.4 at day 28 (Fig. 4(a)–(c)). These precipitates might be considered HCA precursors (octacalcium phosphate), and therefore suggest the capability of NCL7 composition to induce bioactivity [24]. Furthermore, for this silicate-based glass the nucleation of globular precipitates was associated with the formation of micro-cracks (Fig. 4(c)). Crack development is usually a common morphological feature, part of the dual reaction of formation of a silica-rich film and growth of calcium-phosphate HCA layer, which is typical of bioactive materials [1].

Although the NCL2 and NCL7 formulations have similar SiO_2_, CaO, and P_2_O_5_ content, the more complex formulation of NCL2 glass, based on the incorporation of many intermediate oxides, might have reduced its *in vitro* bioactivity, and thus may explain the absence of even HA precursors on its surface [25]. Specifically, the presence of Fe_2_O_3_ and MgO with respect to other bioglasses in SiO_2_ - CaO - P_2_O_5_ system, and their higher content in comparison to the NCL7 main formulation, could have affected the morphology of the precipitates and the NCL2 bioactive process [26], [27].

Globular and flake shaped agglomerates, rich in calcium and phosphorous, were identified on the NCL4 borate-based pellet surface, which increased after 28 days of soaking, as demonstrated by the Ca/P ratio that moved from 0.76 at day 7 (Fig. 3(d)) to 1.08 at day 28 (Fig. 3(f)) leading to the formation of dicalcium phosphate precipitates [24]. Globular agglomerates have been previously observed for less complex borate-based compositions after immersion in SBF [28]; however, for more complex NCL4 composition, the presence of oxides such as MoO_3_ and SeO_2_ might have delayed the formation of the precipitates.

The extensively documented bioactive properties of AW glass-ceramic [29] were further proved in this study. After 7 days in immersion, AW sintered pellets were already completely covered by an HCA layer, which at day 28 reached a Ca/P ratio (Fig. 4(f)) nearly equal to the stoichiometric hydroxyapatite (Ca/P=1.67). It is interesting to observe that although the Ca/P ratio of NCL7 precipitates was lower than AW after 28 days in SBF immersion, conversely the crack formation process was more pronounced on the NCL7 than the AW-derived pellets. As suggested by previous studies [1], [13], [30], this behaviour is considered the initial stage of the formation of the HCA layer on glass and glass-ceramic surface, and hence can be considered a promising feature towards the bioactive potential of NCL7 formulation.

**Fig. 5:**
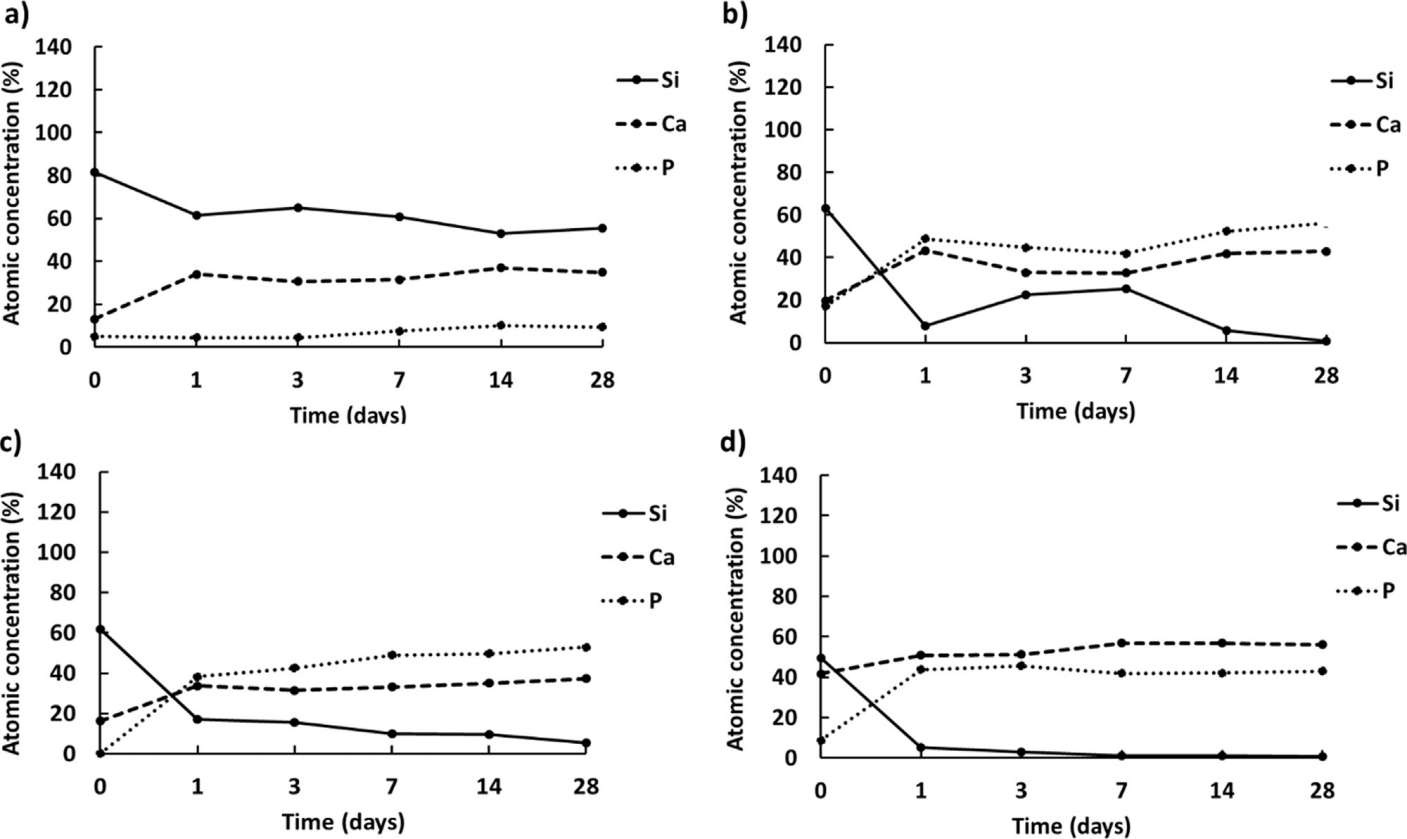
Atomic concentration of Si, Ca and P on the upper surface of a) NCL2, b) NCL4, c) NCL7 and d) AW bioceramic pellets after immersion in SBF solution up to 28 days.

In this study a quantitative elemental characterisation of the sample surfaces before and after soaking in SBF was assessed by XPS analysis, in order to estimate the atomic concentration of the main elements (Si, Ca and P) involved during the bioactivity process [5]. The XPS results (see Fig. 5) evidenced that the atomic concentration of calcium and phosphorous on the surface of NCL4, NCL7 and AW sintered pellets increased already after 24 h in immersion, reaching values higher than silicon and above 40% after 28 days of soaking. It is interesting to note that conversely to AW-based samples (Fig. 5(d)), the level of calcium on the surface of NCL4 and NCL7-based pellets (Fig. 5(b)and (c)) was lower than phosphorous for the entire time interval. This behaviour might, therefore, suggest the presence of HA precursor (as indicated by the EDX analysis), and hence the slower bioactive process for both NCL4 and NCL7 compositions.

Regarding NCL2-based pellets (see Fig. 5(a)), after immersion in SBF solution only the calcium level increased, whereas phosphorous remained almost steady (~9.5 after 28 days in immersion). The lower diffusion of phosphorous from the outermost surface of NCL2-based pellets, in comparison to the other formulations, confirmed the lack of bioactivity of this composition.

### 3.3 pH variation, weight loss and ionic release potential

Usually glass-based structures are known to dissolve in aqueous solutions with a variable rate depending on the kind of network former and its percentage in the glass structure [31].

**Fig. 6:**
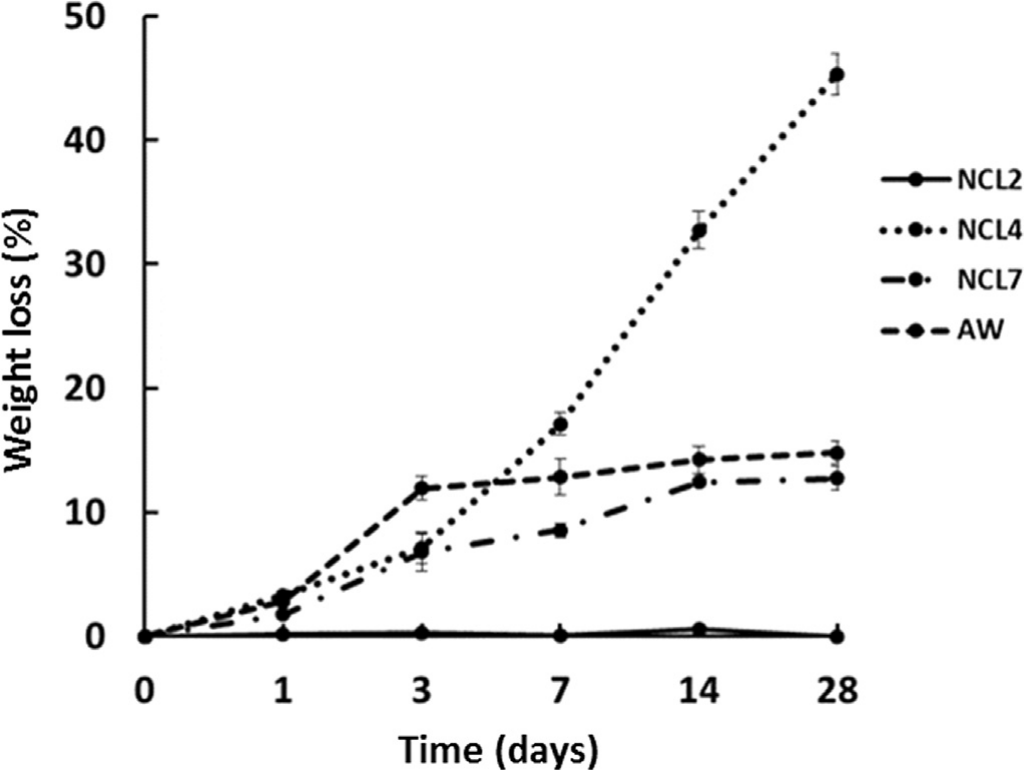
Averaged weight loss (±SD) of NCL2, NCL4, NCL7 and AW pellets after soaking in SBF solution up to 28 days.

For the fabricated pellets, the *in vitro* degradation behaviour was assessed by measuring pellet weight loss after SBF immersion (see Fig. 6). NCL2 silicate-based structures showed the lowest degradation rate all over the considered interval. The negligible weight loss of this class of samples is consistent with the low degradation rate shown by its diopside crystalline phase upon immersion in physiological fluids [22]. NCL7 and AW specimens showed a similar trend upon SBF immersion, reaching a mass loss around 12% and 14% respectively, after 28 days of soaking.

Considering the NCL4 samples, at the beginning small variations were observed; then from 3 to 28 days a greater weight loss was measured. These findings confirmed that borate-based glasses have a faster dissolution rate [32], [33], [34], [35].

On the other hand, the degradation process of a bioactive glass takes place by ionic exchange of soluble ions, which, depending on the glass composition, influence the pH of the surrounding media [36]. In order to evaluate the hydrolytical stability of the bioceramic pellets after immersion in SBF, the pH changes during the 28 days of immersion were assessed.

**Fig. 7:**
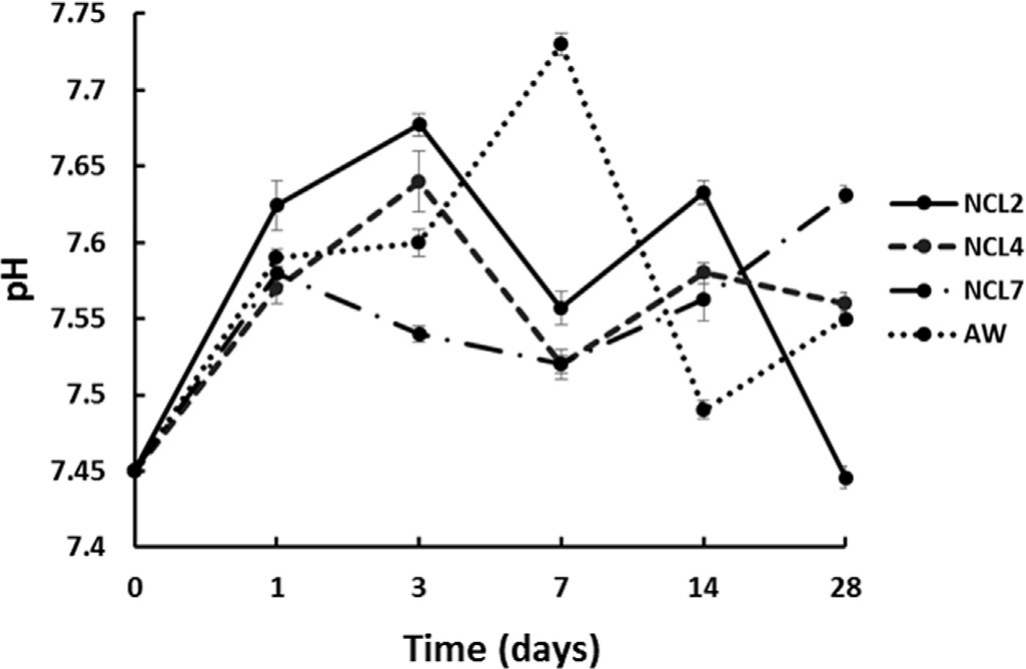
Averaged pH value (±SD) of SBF solution for NCL2, NCL4, NCL7 and AW samples.

Fig. 7 shows the pH variation as function of the soaking time resulting from the solubility/ionic exchange reactions at the solid/liquid interface [37], during the 28 days of immersion in SBF. It can be observed that all the compositions were characterised by a low pH variation over the period, which ranged between 7.44 and 7.74. According to the mechanism proposed by Hench, the pH of the solution rises very fast initially, followed by marginal change in pH with respect to time [5]. In the present case, the almost stable pH values might be governed by the total sum of both basic and acidic ion concentration present in the main composition [27]. pH values around 7 are usually considered optimal in provision of future *in vitro* cell culture [38].

**Fig. 8:**
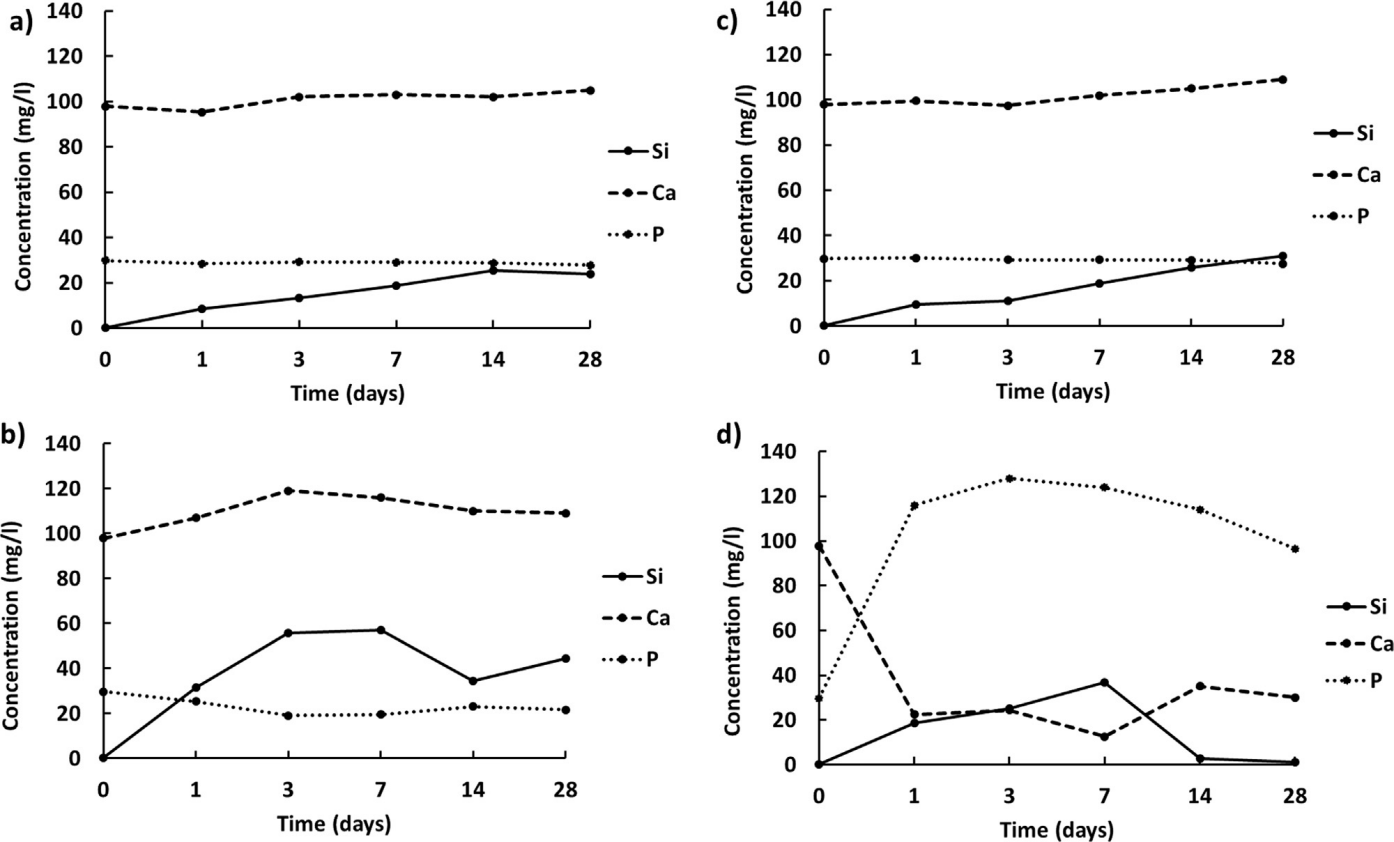
Release profiles of Si, Ca and P ions from a) NCL2, b) NCL4, c) NCL7 and d) AW bioceramic pellets immersed in SBF solution.

In addition, the ionic leaching potential of the sintered pellets soaked in SBF solution was assessed by measuring the amount of Si, Ca, and P released into the media after each time interval. According to the ICP analysis data (see Fig. 8), the highest release of phosphorous occurred for AW composition (Fig. 8(d)), which further proved the bioactivity of this materials [29]. By comparing the silicon leaching profiles for NCL2, NCL4 and NCL7 sintered pellets (Fig. 8(a)–(c)), it is interesting to observe that the presence of boron in the glass structure greatly enhanced the release of this ion. In fact, after 3 days in immersion the concentration of silicon released from NCL4-based pellets reached a value more than 3 times higher with respect to the silicon released from NCL2 and NCL7-based samples. These findings confirm that borate-based glasses have a highly reactive nature [31], [35], [39], and once in solution they can contribute to the dissolution of the other elements present in the glass network.

## 4 Conclusion

Three highly complex glass compositions, NCL2, NCL7 (SiO_2_-based) and NCL4 (B_2_O_3_-based) were sintered in form of dense pellets, following the data derived from HSM analysis. The XRD investigation showed the glass-ceramic nature of NCL2, NCL7 and AW compositions after sintering, whilst the NCL4 formulation remained completely amorphous. The sensitivity of the novel materials on apatite forming ability and biodegradation behaviour was assessed by XRD, SEM, EDS, XPS, ICP and pH variation during 28 days of immersion in SBF. The experimental results have indicated that two similar silicate glass formulations (NCL2 and NCL7) showed markedly different responses in terms of apatite forming ability, microstructure of the precipitates, and dissolution in SBF. The less complex of the two glass formulations, NCL7, exhibited the greatest bioactive potential, while NCL2 showed no apatite precipitates after 28 days in SBF immersion. Furthermore, NCL4 displayed the highest degradation rate, confirming the highly reactive nature of borate-based glass compositions.

In conclusion, the complexity of glass formulations affects significantly *in vitro* bioactivity and degradation behaviour of these materials, as very small variations in glass formulations, (even less than 2 wt%), can change completely their bioactivity and solubility. The combined effect of ions released in aqueous solutions (such as SBF) can enhance or inhibit dissolution and precipitation rates of these materials.
